# Work-Related Musculoskeletal Disorders: A Systematic Review and Meta-Analysis

**DOI:** 10.3390/jcm13133964

**Published:** 2024-07-06

**Authors:** Chiara Greggi, Virginia Veronica Visconti, Marco Albanese, Beatrice Gasperini, Angela Chiavoghilefu, Caterina Prezioso, Benedetta Persechino, Sergio Iavicoli, Elena Gasbarra, Riccardo Iundusi, Umberto Tarantino

**Affiliations:** 1 Department of Clinical Sciences and Translational Medicine, University of Rome Tor Vergata, Via Montpellier 1, 00133 Rome, Italy; chiara.greggi@gmail.com (C.G.); elenagasbarra@tiscali.it (E.G.); riccardo.iundusi@uniroma2.it (R.I.); umberto.tarantino@uniroma2.it (U.T.); 2 Department of Biomedicine and Prevention, University of Rome Tor Vergata, Via Montpellier 1, 00133 Rome, Italy; beatrice.gasp95@gmail.com; 3 Department of Statistics, University of Rome Tor Vergata, 00133 Rome, Italy; marco.albanese@uniroma2.eu; 4 Department of Orthopaedics and Traumatology, “Policlinico Tor Vergata” Foundation, Viale Oxford 81, 00133 Rome, Italy; angela.chiavo@hotmail.com (A.C.); caterina.prezioso94@gmail.com (C.P.); 5 Italian Workers’ Compensation Authority (INAIL), Monte Porzio Catone, 00078 Rome, Italy; b.persechino@inail.it (B.P.); s.iavicoli@inail.it (S.I.); 6 Faculty of Medicine, University “Our Lady of Good Counsel”, 1000 Tirana, Albania

**Keywords:** musculoskeletal disorders (MSDs), work-related activities, healthcare, farming, industrial, computer

## Abstract

**Background:** Musculoskeletal disorders (MSDs) involve muscles, nerves, tendons, joints, cartilage, and spinal discs. These conditions can be triggered by both the work environment and the type of work performed, factors that, in some cases, can also exacerbate pre-existing conditions. This systematic review aims to provide an overview of the impact that different work-related activities have on the musculoskeletal system. **Methods:** A global search of publications was conducted using the following international bibliographic web databases: PubMed and Web of Science. The search strategies combined terms for musculoskeletal disorders and workers. In addition, a meta-analysis was conducted to estimate the prevalence of MSDs within the healthcare sector. **Results:** A total of 10,805 non-duplicated articles were identified, and finally, 32 studies were reviewed in this article. Once the literature search was completed, occupational figures were categorized into healthcare, farming, industrial, and computer sectors. In the healthcare sector, the prevalence estimate for degenerative diseases of the lumbar spine was 21% (497 out of 2547 physicians and dentists) (95% CI, 17–26%), while for osteoarthritis of the hand, it was 37% (382 out of 1013 dentists) (95% CI, 23–51%). **Conclusions:** Musculoskeletal disorders significantly impair workers’ quality of life, especially in healthcare sector. These conditions are also associated with high costs for employers, such as absenteeism, lost productivity, and increased costs for healthcare, disability, and workers’ compensation.

## 1. Introduction

Among the leading causes of long-term disability and illness are musculoskeletal disorders (MSDs), which are closely related to functional disability and, consequently, to high expenditure on health and social resources [1]. The musculoskeletal system represents the fundamental structural component of the body, comprising muscles, bones, joints, and connective tissues, the continuum of which is represented by the fascial system that is able to envelop, interpenetrate, and support bone tissue and skeletal muscles [2,3]. The health of this complex system is linked to the performance of its components, the alterations of which underlie approximately 150 different pathological conditions [4]. Musculoskeletal disorders are typically characterized by pain and temporary or lifelong limitations in mobility and dexterity, which reduce people’s ability to work and participate in social life [5]. To date, work-related MSDs are among the major risk factors for the occurrence of certain diseases, such as osteoarthritis, osteoporosis, and sarcopenia, and conditions that also affect multiple body areas or systems, such as a regional pain state and inflammatory diseases [1,6]. These pathological conditions are considered major diseases affecting millions of workers, resulting in a high cost of billions to companies and public health systems [7]. A common aspect of these conditions is also represented by work-related pain, which can affect different body districts. Among these, the hand certainly represents the site most involved, especially in the context of professional activities that require accurate and repetitive movements and the maintenance of specific positions for prolonged times [8,9]. Several factors contribute to the onset and progression of these disorders, including predisposing genetic factors and environmental factors [10]. The latter include workplaces and the activities that are performed in these settings, where we spend most of our daily lives. This systematic review aims to provide a comprehensive overview of the impact that different work-related activities have on the musculoskeletal system. The most prevalent MSDs among occupational groups representing the general population were considered. Hence, occupations were classified into healthcare, farming, industrial, and computer sectors.

The main objective of this study is to highlight which musculoskeletal health problems result from inadequate and compromising work activities in the general population. To better investigate this topic, we conducted a literature review up to March 2022, without time filters. At the end of the search, we divided the selected works into four occupational categories that best represent the population of workers worldwide: healthcare, farming, industrial and computer sectors. This topic reflects a worldwide public health problem that entails significant costs for employers, hospitals, and individuals. It seems crucial to better frame this issue and its causes, especially in the workplace, given the seemingly unstoppable growth trend of MSDs, in order to develop effective and lasting prevention strategies. 

## 2. Materials and Methods

### 2.1. Literature Search Strategy and Eligibility Criteria

The systematic review was conducted in accordance with the general principles recommended in the statement Preferred Reporting Items for Systematic Reviews and Meta-Analyses (PRISMA) [11]. The complete search protocol is provided in Supplementary Materials (Table S1). It was also registered prospectively with PROSPERO (ID registration: CRD42022336630). The PubMed and Web of Science databases were searched using the following keywords and boolean operators: ‘((MUSCULOSKELETAL SYSTEM) AND (WORKERS))’. The investigation to identify papers relevant to our issue was carried out up to 25 March 2022. The search was performed without restrictions on ethnicity or geographic areas. Language and species filters were then applied to the list of output results to eliminate non-English articles. The first selection of studies was made on the basis of the consistency of the title with the research topic, and subsequently, articles were selected on the basis of the abstract. The articles were independently screened by two of the authors, and the controversial issues were resolved in a debate. Systematic reviews, meta-analyses, RCTs, and original works whose full text was available were included. Otherwise, narrative reviews, case reports, studies involving animal models, in vitro studies, cadaver studies, and studies based on self-assessment questionnaires were excluded. Excluded job categories were military personnel, firefighters, dancers, musicians, airplane pilots, and drivers. Finally, all works related to musculoskeletal pain were excluded. The primary endpoint of this study is estimating the prevalence of MSDs related to different occupational activities in the four identified sectors.

### 2.2. Data Extraction and Quantitative Synthesis

Data from each study reporting prevalence estimates were extracted. These included the following: study design, occupation, sample size, the mean age of physicians and dentists, and reported prevalence of work-related MSDs. Meta-analysis was performed using a random-effects model. Statistical heterogeneity was assessed with the I^2^ statistic. Weighted proportions and their 95% CIs were summarized in forest plots. R software was used for the statistical procedures (R, Version 4.3.1 16 June 2023; R Foundation for Statistical Computing, Vienna, Austria).

## 3. Results

### 3.1. Study Search and Study Characteristics

**Figure 1:**
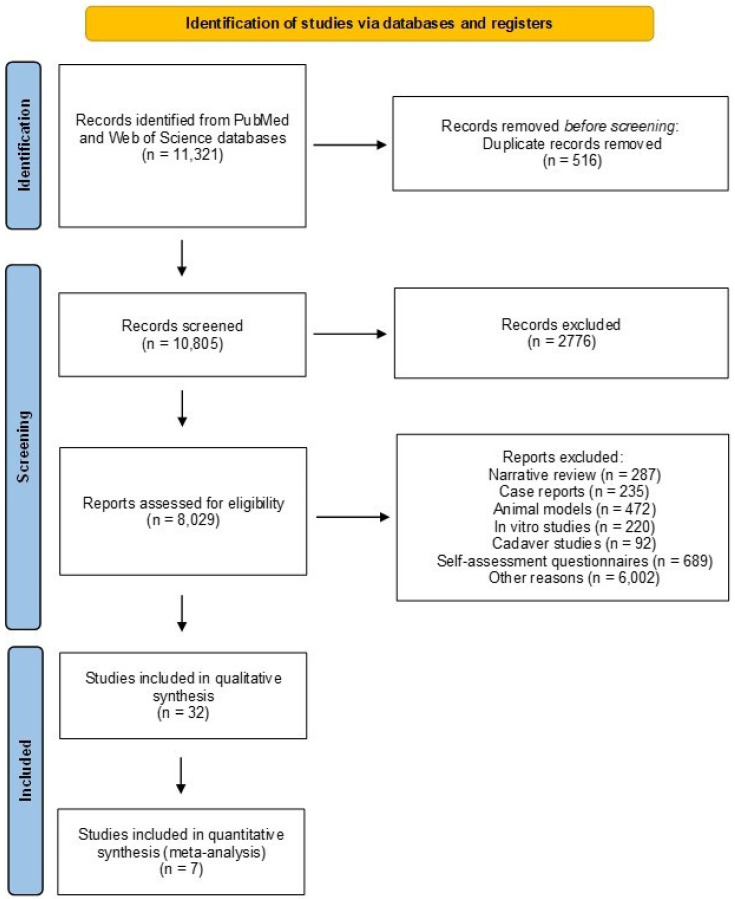
PRISMA (Preferred Reporting Items for Systematic Reviews and Meta-Analyses) flow diagram of the search strategy and outcome in the review.

Out of 11,321 papers identified in the database searches, a total of 10,805 non-duplicated articles were screened, and finally, 32 papers met the inclusion criteria for the qualitative analysis (Figure 1). In fact, the primary studies included cross-sectional studies and a single systematic review and meta-analysis. Studies correlating impaired musculoskeletal health with prolonged work activities were described and categorized into four areas: healthcare, farming, industrial, and computer sectors (Table 1). For the purpose of performing quantitative data analysis, the most homogeneous studies were selected, i.e., studies in which the following information was clearly reported: professional activity performed, pathology of interest, the mean age of the study population, and prevalence of the pathology of interest. As a result of this selection, seven studies were eligible for meta-analysis. Specifically, seven studies from the healthcare sector showed eligibility for the meta-analysis of the prevalence of MSDs, including degenerative lumbar spine disease and hand osteoarthritis, among at-risk workers (Figure 1).

### 3.2. Healthcare Sector

According to the literature, physicians involved in specific procedures, such as surgeons or interventional medicine specialists, are found to have a high risk of MSD occurrence (Table 1). A meta-analysis reports crude prevalence estimates of the most common work-related MSDs in physicians, including degenerative cervical spine disease in 17%, rotator cuff pathology in 18%, degenerative lumbar spine pathology in 19%, and carpal tunnel syndrome in 9% [6]. Failure to engage in regular physical activity resulted in part of an erroneous lifestyle that appears to characterize this category: due to irregular and hectic work schedules, this professional group leads, in fact, to a sedentary lifestyle, has poor dietary habits, and receives low exposure to sunlight—risk factors that greatly affect the health of the musculoskeletal system. Multani and colleagues report how healthy young medical residents from a tertiary referral center in Mumbai were characterized by significantly lower bone mineral density (BMD) than the healthy Indian population. In this study, osteopenia was found in 59.7% of males and 67.5% of females, while 18.39% of males and 12.5% of females had osteoporosis [12]. The positive contribution of physical activity on musculoskeletal system health status has also been demonstrated by Weiss et al., who analyzed the hip BMD of hospital clerks compared with that of nurses. Using dual-energy X-ray absorptiometry (DXA), in fact, higher femoral BMD was found in the nurses’ group than in the office workers, which correlated positively with serum osteocalcin levels and the duration of work time spent standing, a type of exertion that has been described as more osteogenic [18]. Among various professional categories, dentistry imposes constraints on the musculoskeletal system: this profession requires considerable bimanual work, the performance of repeated arm and hand movements over prolonged periods, and a precision grip to handle certain instruments. In addition, this work involves taking uncomfortable postures for the neck and upper limbs, requiring the contraction of more than 50% of the body’s muscles to keep them in a static position [43]. In 2006, Solovieva et al. showed that the pattern of work activity was closely related to the occurrence of osteoarthritis (OA) in the thumb, index, and middle finger joints among female dentists: in fact, dentists with a history of low variation in hand movements had a higher prevalence of OA in the thumb, index, and middle fingers compared to dentists with high variation. This work, therefore, suggests how low variability in movements is a main risk factor for the development of this pathology in this occupational category: avoiding monotonous work activities could, therefore, reduce the risk of finger OA in the dentists’ category [14]. Later, Ding and colleagues compared the occupational category of dentists with that of teachers, two occupations characterized by different applied load on the hand. In this work, it is reported that for both categories, the overall prevalence of symptomatic hand OA was 10.1% in the left hand and 12.2% in the right hand, although no difference in prevalence was found between the two categories. Furthermore, it was shown that symptomatic OA was associated with the risk of reduced grip strength in both occupations, suggesting how the severity of OA impairs hand function in these occupational categories [13]. The results of this study agree with previous results from the same research group. Indeed, in this study, the two occupational categories are again compared, and it is highlighted how the two occupations are primarily characterized by different patterns of joint involvement. The overuse of these joints has been associated with varying degrees of risk for the onset of finger OA. Specifically, the odds ratio (OR) for more severe OA (grade 3 or higher) in the right thumb, index, and middle fingers was significantly higher among dentists compared to teachers [15].

What was reported above finds support in the work of Lehto and colleagues, in which it was observed that arthrosis of the distal interphalangeal (DIP) joints occurs in a higher proportion in the experimental group of dentists compared to healthy controls, especially under the age of 50, suggesting that arthrosis of the DIP joints of the fingers develops earlier in dentists than in the general healthy population [17]. The dentistry category can be subject to high physical labor; thus, the dentist’s profession is also characterized by spinal MSDs. In a study by Katevuo et al., this occupational category was compared with that of farmers. This study reports how 52.1% of dentists were affected by cervical spine spondylosis, compared to 19.2% of farmers, highlighting how this occupational category can be considered subject to heavy work [16].

Therefore, according to the studies analyzed, the prevalent diseases are osteopenia in the medical category and osteoarthritis of the hand in the dental category. In the first case, lifestyle is a possible underlying cause of MSD, while in the second case, the above-mentioned pathology could be due to repetitive and prolonged hand movements (Table 2).

#### Metanalysis and Quantitative Synthesis

**Figure 2:**
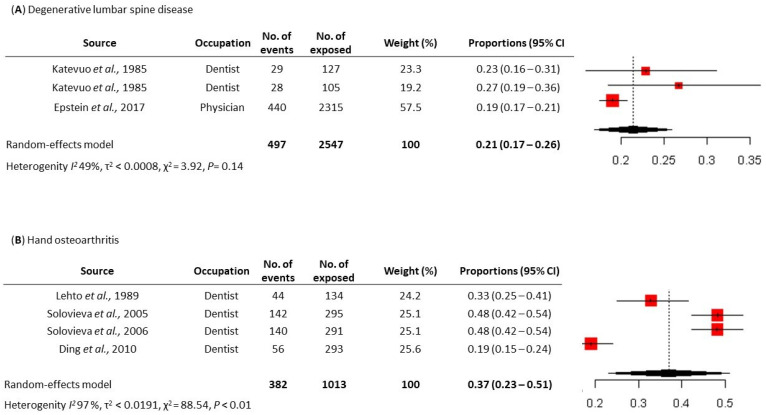
Meta-analysis of the prevalence of work-related Musculoskeletal Disease (MSDs) among at-risk workers in the healthcare sector. (**A**) Prevalence of degenerative lumbar spine disease [6,16] and (**B**) prevalence of hand osteoarthritis [13,14,15,17].

In the healthcare sector, seven primary studies were eligible for meta-analysis. This survey revealed that the prevalence of degenerative diseases of the lumbar spine was 21% (497 out of 2547 physicians and dentists) (95% CI, 17–26%) for dentists and physicians, while the prevalence of osteoarthritis of the hand was 37% (382 out of 1013 dentists) (95% CI, 23–51%) for the professional category of dentists alone (Figure 2). We observed greater heterogeneity in the studies investigating the prevalence of osteoarthritis of the hand in the dentist’s occupational category, probably related to the study by Ding and colleagues, Which reported a very low prevalence (19%) for this condition compared to other studies [13].

### 3.3. Farming Sector

As an occupation, farming requires enormous physical labor. At all body sites, 60% to 92% of farmers have at least one MSD (Table 1) [44,45]. The prevalent pathologies are osteoarthritis of the knee and hip and osteoporosis. Probable causes could be maintaining uncomfortable postures for prolonged periods, lifting excessive loads, a sedentary lifestyle, and working excessively long shifts (Table 2). Dairy farm workers are particularly at risk of developing OA of the knee due to uncomfortable postures, lifting heavy loads, kneeling, and squatting [46]. In an interesting study by Nonnenmann et al., two different milking modes were compared: the stanchion facility, which corresponds to traditional barn milking mode, and the parlor facility, which involves performing the activity in the milking parlor to investigate possible causes of knee OA in this occupational category. Specifically, it was observed that the parlor facility results in reduced musculoskeletal load exerted on the knee compared to the stanchion facility. In fact, the traditional milking mode requires more manual techniques, in which the operator has to stoop and squat to perform the milking, whereas, in a parlor facility, the operator is at a lower level than the animal and is therefore not required to stoop or squat. According to reports in this study, milking parlor workers were less exposed to uncomfortable knee posture, which involves ≥70° and ≥110° bending. In contrast, during traditional stall milking, operators flexed the knee ≥70° 22% of the time and ≥110° almost 18% of the time. Knee flexion ≥110° was indeed identified as a cause of increased compressive forces on the knee, suggesting that in this occupational category, this particular way of performing work may be a cause of OA [19]. There are numerous studies in the literature investigating different types of agricultural work in relation to the development of OA of the knee and the hip. In the study by Thelin and colleagues, a group of 427 farmers with OA of the hip and a control group of healthy farmers were compared; this investigation showed that farmers who ran farms characterized by a large area of crop production that did not involve direct, long-term contact with animals had a significantly lower risk of developing OA of the hip compared to farmers in general. This study is, therefore, in agreement with what Nonnenmann and colleagues stated, as working in contact with animals results in poor postures that have deleterious effects on the joints, compromising the health of the musculoskeletal system [20]. MSDs affecting the lower limbs are found with frequency among rice farmers; in fact, according to literature data, rice farmers are characterized by a 34.9% prevalence of knee MSDs, causing leg pain, reduced mobility and work capacity, and overall, significant physical disability [47]. Specifically, as reported by Puntumetakul et al., rice farmers have the highest prevalence of impairments of the knee joint (79.6%), followed by impairments in the hamstring muscle (52.74%), quadriceps muscle (44.28%), neural tissue (38.81%), knee ligaments (1.99%), and meniscus (1.49%) [21]. In addition, factors associated with the highest prevalence of these disorders include female sex, age > 40 years, being overweight, farming for more than 10 years, and working for more than 5 h a day [48,49]. The workload seems to exert effects not only on joint health status but also on BMD, as already mentioned for health professions. In fact, Cho et al. showed how women working in agriculture exhibit altered bone metabolism compared to women in non-agricultural occupations. Indeed, in this study, lower femoral neck BMD was found in women working in agriculture compared to the control group, in parallel with increased levels of bioavailable 25-hydroxy vitamin D (25(OH)D) and decreased levels of vitamin D transporter, vitamin D-binding protein (VDBP). In addition, the risk of developing osteoporosis was calculated for both experimental groups, which was found to be 4.3 times higher for women in agricultural work [22]. Due to the nature of agricultural work, upper limb MSDs, on the other hand, are particularly common among fruit tree growers, who usually work while maintaining a static position, with their arms and shoulders raised, repeatedly moving their hands to pick fruit. Indeed, in the study by Kim and colleagues, it is reported that the most common MSDs in this occupational category are rotator cuff tear (60.4%) and hand OA (58.0%). Again, women were found to be more vulnerable to the occurrence of these disorders: according to literature studies, male fruit tree growers worked an average of 10.2 h per day during the agricultural season and 5.2 h per day during the non-farm season, while female fruit tree growers worked an average of 10.6 h per day during the agricultural season and 5.5 h per day during the non-farm season [23].

### 3.4. Industrial Activities

Literature data identify strong correlations between musculoskeletal system disorders and different occupational activities performed by classes of workers in the textile, construction, and handicraft industries (Table 1). These different work activities involve postures and movements that can affect bone and muscle health, such as equipment vibration, kneeling, loading of weights, and continuous manual clenching activities [50]. Vibration-exposed workers are mainly represented by foundry workers, and the effect of this activity was investigated in a study of 67 foundry workers exposed to vibration and 46 reference subjects who performed heavy manual work [24]. The aim is to study the prevalence of musculoskeletal symptoms and radiographic abnormalities in the wrists, elbows, and shoulders. Both the radiological signs of osteoarthritis in the wrist joint and the overall prevalence of radiographic abnormalities in the elbow joint occurred more frequently in the group subjected to vibration, showing olecranon spurs in 50.7% of the exposed subjects [24]. These results show that foundry workers using vibrating tools are affected by bone and joint disorders in the elbow and wrist. Elbow joint disorders in relation to vibration exposure were also studied in 74 male stone quarry workers who used cutting hammers and sometimes rock drills [25]. This study demonstrated a decreased range of motion in the right elbow in relation to the duration of vibrating tool use, showing more severe OA with increasing age and duration of vibrating tool use [25]. Exposure to hand-arm vibrations could be a major risk factor for the back and neck, especially for miners, posing a public health problem. A study that analyzed 685 X-ray films of the cervical spine from underground coal mine workers in Poland investigated the presence of narrowed disc spaces and osteophytes in this occupational group and examined the association of radiographic changes with age, duration of work, and duration of occupational exposure to hand-arm vibrations [26]. Narrowed intervertebral disc spaces were found in 26.9% of coal miners and osteophytes in 47.5% of coal miners. Narrowed intervertebral disc spaces and osteophytes were more frequent among older individuals with longer durations of employment. However, when both radiographic changes were pooled, age remained the only statistically significant explanatory variable. Thus, the definitive results do not support the existence of a correlation between the degenerative changes identified in the cervical spine in coal miners and the duration of physical work or exposure to hand-arm vibrations in this occupational category [26]. Another occupational category exposed to hand-arm vibrations is represented by tire specialists, in which the association between vibration exposure and hand-arm vascular syndrome (HAVS) is investigated [27]. This study involved 200 male workers in a tire workshop, divided according to the time of vibration exposure, in whom HVAS was assessed with a vibration meter. The high-exposure group showed a higher prevalence of musculoskeletal complications. Despite the significant difference in musculoskeletal complications between the two exposure groups, there was no significant difference in the stratification of musculoskeletal complications of the upper limbs and neck. Only hand grip weakness showed a significant difference between the two exposure groups. However, it is also necessary to consider other important factors, such as ergonomics, grip strength, and posture, which may contribute to the development of musculoskeletal complications associated with HAVS [27]. Vibrations, together with other factors such as awkward posture, lifting heavy loads, high force, cold, mechanical presses, improper tool use, and localized stresses, are interconnected with MSDs of the upper limb. An interesting study analyzed 80 coal miners and 43 employees of the same age to evaluate tendinitis and entrapment neuropathy of the upper limb among Turkish coal miners. The coal miner group had a statistically significant difference in the prevalence of lateral epicondylitis and De Quervain’s disease. Ulnar neuropathy of the elbow (UNE) was also very common in this occupational group, showing a statistically significant increase compared to the control group. This study demonstrates a strong correlation between the occurrence of these three upper limb disorders and the work activity of coal miners [28]. The hypothesis that manual work and vibration exposure are antecedents to the development of osteoarthritis was addressed in a study that included three groups of manual and non-manual workers from the construction industry. The results showed that construction workers who lifted more than 709 tonnes had an increased risk of developing severe OA of the right acromioclavicular joint. However, vibration alone did not explain the clinical phenotype analyzed, and other factors, such as the sum of tonnes lifted during working life, may also be risk factors for acromioclavicular joint OA [29]. The influence of work and physical activity would appear to play a role in the rate of degenerative change of the pubic symphysis in Portuguese males. This study analyzed a total of 161 male workers, divided into manual and non-manual work and further categorized into robust and gracile groups. The influence of occupational activity on the rate of aging showed a faster rate of change of ligamentous outgrowths on the ventral bevel in individuals with more physically demanding work activities [30]. The focus on workload was also placed on identifying this mechanical stressor as a risk factor for lumbar disc degeneration. In this case, the subjects studied included 53 machine drivers, 51 construction carpenters, and 60 municipal office workers. Magnetic resonance imaging (MRI) showed an increased risk of posterior disc bulges among carpenters and anterior disc bulges among machine drivers. Driving a car also appeared to be associated with anterior disc bulges [31]. Disc degeneration in the lumbar spine has also been observed in foundry workers [32]. A study of 325 workers from 10 different foundries identified a lower incidence of rheumatic disorders compared to a random sample, probably due to the higher heat exposure experienced by these subjects. However, radiological evidence of disc degeneration in the lumbar spine appeared more frequent in foundry workers, who also showed greater severity of the phenotype [32]. The upper limbs can be affected by both heavy dynamic and prolonged static occupational activities. Specifically, in a study comparing welders, office workers, and fishermen, it was shown that atrophic shoulder muscles were more common among welders than fishermen, while shoulder crepitations tended to be more common among fishermen. These data indicate that both types of work can induce shoulder injuries but of different types [33]. Prolonged work-induced kneeling is also a condition that can lead to musculoskeletal disorders, such as an increased risk of injury or degenerative diseases of the knee joint. This is especially true for coal miners, who are subjected to constant work-related kneeling [34]. Knee work was also evaluated in association with degenerative meniscal tears in floor workers and graphic artists undergoing MRI. This study showed a more significant prevalence of degenerative tears and a higher number of medial tears in both knees among floor workers compared to graphic artists. These data support the hypothesis that occupational kneeling contributes to the risk of developing degenerative tears of the medial menisci, but not the lateral menisci, in both knees [35]. Knees can also be subjected to work-related loads, such as in concrete reinforcement and maintenance painting work. A study of 352 workers showed that loads on the knees and the occurrence of minor injuries and accidents were higher in reinforcement work compared to painting, but symptoms, clinical signs, and radiological findings were equally common in both groups [36]. Occupational squatting is also correlated with bone health quality parameters in a study analyzing the relationship between occupational activities and bone health quality parameters in 158 out of 165 women in India, including beedi (cigarette) makers, sweepers, and construction workers [37]. The BMD of the femoral neck and hip did not differ among the three groups, but the femoral neck bone area was higher in beedi makers compared to sweepers, probably due to the squatting position adopted by beedi manufacturers. The BMD of the lumbar spine was significantly lower among sweepers compared to beedi manufacturers, and the groups performing walking and loading activities (sweepers and construction workers) had a higher prevalence of osteoporosis in the lumbar spine [37]. These results might suggest that bone parameters may be influenced by both the different types of activity, either loading or squatting, and the phenomenon of undernutrition typical of developing countries. Another study addressing the assessment of bone mineral density in workers from non-industrialized countries was conducted on 200 women from garment factories in Bangladesh [38]. About 16% of the subjects were found to be deficient in vitamin D, which was associated with a progressive reduction in bone mineral density at the femoral neck and lumbar spine. Subjects with a bone mineral density T-score < −2.5 tended to have lower BMI values, waist-to-hip circumference, mid-upper arm circumference, and serum 25-hydroxyvitamin D (S-25OHD) levels, showing a correlation between indoor lifestyle, low dietary calcium intake, and bone mineral status [38]. In the industrial sector, prevalent diseases are knee and upper limb osteoarthritis, osteoporosis, upper limb and neck disorders, and lumbar disc degeneration. These conditions are potentially due to maintaining uncomfortable and static postures, vibrations caused by the use of certain machinery, performing repetitive movements, and lifting excessive loads (Table 2).

### 3.5. Computer Workers

The age of technology has led to a general increase in computer use, concomitant with a significant increase in the number of people with musculoskeletal health problems, particularly in the upper limb and neck (Table 1) [51,52]. Specific and nonspecific symptoms reported by individuals who routinely use computers are variously referred to as repetitive strain injury (RSI), cumulative trauma disorder (CTD), or work-related upper extremity disorder (WRUED) [39]. Nevertheless, all these designations do not attribute the cause of musculoskeletal signs exclusively to repetitive motion due to computer use during work activity but also to biomechanical or psychosocial stress factors. One study showed how mouse and keyboard users could contribute to neck, shoulder, and upper extremity disorders [53]. This may be explained by the device’s distance from the body’s midline, which leads users to work with their arm unsupported, with the shoulder externally rotated and the arm in forward flexion [54]. Burgess and colleagues, in their study, measured maximum wrist flexion using a protractor, with the forearm in supine, neutral, and prone positions. The enrolled five subjects who habitually used computers reported MSDs of the upper extremity, and 13 control subjects had no symptoms and did not use computers. The computer users appeared to show limited wrist flexion, attributed to increased passive tension in the wrist extensor muscle, particularly the extensor carpi ulnaris, a muscle primarily responsible for wrist extension or reduction in the angle between the back of the hand and the forearm. Upper extremity musculoskeletal disorders (UEMSDs) subjects never reported experiencing pain, demonstrating that the decrease in wrist flexion was not limited by pain [39]. These data were confirmed using an experimental case-control study measuring cervical postural muscle load with surface electromyography (EMG) in the group of the cases and controls. Cases were symptomatic women with moderate to severe neck and upper extremity discomfort for at least 3 of the past 12 months. Those with mild discomfort for less than 3 months and no pain were assigned to the control group. The analysis showed that symptomatic subjects tended to perform computer work with more significant muscle load than non-symptomatic subjects. This could represent a mechanism underlying the development of symptoms and an appropriate cue for physiotherapeutic management [40]. Mouse use, compared with keyboard use, requires higher visual input, which results in increased activity in the neck muscles [55]. Based on these data, Arvidsson and colleagues investigated whether the musculoskeletal health of air traffic controllers was affected by switching from moderate-intensity computer work to high-intensity computer work with predominantly mouse use. In all subjects, neck and upper extremities were examined before and about 20 months after the switch. The switch from moderate computer work to a mouse-only system caused a significant change in the workers’ physical exposure. The results showed a significant increase in elbow and hand discomfort in both young and old workers. This work aims to emphasize that preventive measures need to be implemented, such as the use of short keyboard commands instead of a mouse, to reduce risks to the musculoskeletal system [41]. A further study confirmed that mouse use could negatively impact musculoskeletal health and lead to impaired muscle activity. Using EMG demonstrated that individuals with computer-related wrist/hand symptoms showed altered motor control mechanisms in forearm muscles that contribute to work-related MSDs [42]. Further studies are needed to better understand musculoskeletal disorders’ occurrence in workers who employ portable video display units as an elective tool. Unfortunately, the literature data are still full of gaps. Expanding the case series and better characterizing experimental groups could help to fully understand the impact of these electronic devices on musculoskeletal health. In this sector, the most common MSDs are those affecting the upper limbs, neck, and shoulders. These conditions are potentially due to performing repetitive movements, assuming a certain posture for a prolonged time, and the distance of the device used from the body’s medial axis (Table 2).

## 4. Discussion

Occupation-related MSDs represent all those conditions of physical discomfort involving bones, muscles, ligaments, and nerves that occur in performing some occupational activities. Such disorders can affect workers from different sectors and occupations, representing a significant problem for the overall healthcare system [56]. Our systematic review of the literature led to the selection of 32 primary studies, adopting corrective strategies by two independent operators to ensure the quality of the included studies. The primary studies selected consisted of cross-sectional studies, systematic reviews, and meta-analyses, which were divided into four areas: healthcare, farming, industrial, and computer sector. The causes can be diverse and include, separately or in combination, both physical factors, including vibration, force, awkward postures, repetition, and environmental factors, such as dark workplaces, long working hours, and psychosocial characteristics. In order to highlight the most frequent pathologies in the described occupational sectors, we have listed the occupational categories related to each area and the musculoskeletal disorders most represented in each category, also indicating the plausible causes underlying the occurrence of these disorders. Interestingly, it is possible to identify plausible causes that are differentially spread among the four occupational sectors, such as vibrations, which are the most common cause of several MSDs in workers belonging to the industrial sector, along with the assumption of awkward postures during the performance of occupational movement in this same category [57]. On the other hand, plausible causes of MSDs in the dentist category include repetitive precision movements performed over a prolonged period [58]. The quantitative analysis showed that osteoarthritis of the hand has a higher prevalence than degenerative diseases of the lumbar spine in the healthcare sector. Specifically, osteoarthritis of the hand was found to have a prevalence of 37% among the professional category of dentists, while degenerative diseases of the lumbar spine showed a prevalence of 21%. Regarding the statistical analysis conducted to calculate the prevalence of osteoarthritis of the hand, the heterogeneity of the studies was greater, probably due to the fact that in the study by Ding and colleagues, the diagnosis of osteoarthritis was made on the basis of the co-occurrence of pain and radiographic signs in the wrist and joints of the first, second, and third fingers, while in the remaining three papers, the diagnosis was made only on the basis of the radiographic report with regard to the status of the interphalangeal joints. In fact, the most recent studies confirm what has been reported in this paper, namely that in the healthcare sector, surgeons and dentists represent the professional category with the highest prevalence of MSDs affecting the back, shoulders, and upper limbs [59]. Similarly, even in the occupational category of office workers, the most common MSDs are upper-body MSDs for the same reasons. In addition, the prevalence of computer work-related MSDs increased during the COVID-19 pandemic and appears to be related to remote work [60]. The solution to this issue could be to correct uncomfortable postures assumed during work activities through the use of ergonomic solutions while also adapting the work environment, with the aim of reducing MSDs according to the specifics of each profession [59]. In addition, for office workers, early self-assessment of MSDs has been shown to be critical in preventing severe symptoms and long-term consequences [61]. The prevalence of MSDs in the agricultural sector is also currently high, and according to this previous data, the main cause is always the postures assumed, which are really dangerous for the health of the musculoskeletal system. In fact, the working hours are too long, and the tasks performed are too repetitive, with the adding psychosocial factors, such as pressure to complete work within a set time [62,63]. Interestingly, workers in the industrial sector are also currently monitored for occupational risks and musculoskeletal disorders. A recent cross-sectional study shows that the most common abnormalities in this category of workers were tendinopathies and intervertebral disc disorders, and the most common risk factors were always manual work (96.7%), 8-h working days (80%), and repetitive gestures (86.7%) [64]. Given the socio-economic burdens that MSDs entail, early diagnosis of these disorders is essential, concurrently with improving working conditions underlying the onset of these conditions [65]. In addition to early diagnosis, it is also crucial to develop effective prevention strategies to prevent these disorders from occurring. According to reports by da Costa and colleagues, performing stretching scise could prevent the onset of these disorders, as the physiological effects of stretching are increased range of motion (ROM), short-term pain relief, and changes in the elastic properties of the muscle–tendon unit. However, it must be kept in mind that only certain occupational categories might benefit from this practice, and if performed inadequately, stretching might further compromise the health of the musculoskeletal system [66]. Moreover, a key prevention strategy is based on ergonomics, which adapts workplace conditions and work demands to fit the capabilities of the general working population, with the goal of reducing the negative impact of certain work activities on the musculoskeletal system. An appropriate ergonomic approach can prevent bone and muscle disorders by eliminating or reducing workers’ exposure to risk factors, such as awkward postures, repetition, material handling, force, mechanical compression, vibration, extreme temperatures, glare, inadequate lighting, and duration of exposure, using engineering and administrative controls [67].

Although this study represents a detailed review of the literature over a very long-time span on a topic of major public health interest, it unfortunately has limitations. The primary studies selected are characterized by considerable heterogeneity in each of the four areas, dating back to different time periods. This, therefore, made it difficult to carry out a more extensive meta-analysis, which was, in fact, only conducted on seven studies that were suitable for quantitative evaluation.

## 5. Conclusions

This systematic review identified four main occupational areas related to MSDs: healthcare, farming, industrial, and computer sectors. Each of these categories is characterized both by a defined pattern of movements performed repetitively by the musculoskeletal system, by the assumption of awkward and incorrect postures for prolonged periods of time, and by the different stresses to which the body is subjected. Despite the different work contexts of the four occupational categories, work-related MSDs are mostly represented by degenerative joint diseases, specifically osteoarthritis of the knee, which is especially prevalent among the occupational category of farmers, but also by disorders affecting the upper limbs, in most cases of which the shoulder in industrial workers is represented. Identifying the factors underlying the occurrence of these conditions is therefore crucial for the prevention of these disorders in occupational categories, and therefore, the purpose of this systematic review is to provide a solid evidence base for researchers and national occupational safety and health communities in their task of preventing work-related MSDs.

## Figures and Tables

**Table 1:** Characteristics and details of 32 included studies.

Outcome	Diagnostic Technique	Occupational Category	N° of Subjects	Age (Mean ± SD or Range)	N° Male, N° Female	Reference
Healthcare Sector						
Osteopenia in 59.7% males and 67.5% females; osteoporosis in 18.39% males and 12.5% females	Dual-energy X-ray absorptiometry, Parathyroid hormone, and 25(OH)D assay	Resident doctors	214	M: 26.87 ± 1.60; F: 26.33 ± 1.58	M: 174; F: 40	[12]
Symptomatic hand osteoarthritis	Pinch grip strength and hand radiographs	Dentists; Teachers	543	Dentists:54 ± 6; Teachers: 54 ± 4	F: 543	[13]
Osteoarthritis in the thumb, index, and middle fingers	Hand radiography	Dentists	291	54 ± 6	F: 291	[14]
Odds Ratio for more severe OA (grade 3 or higher) in the right thumb, index, and middle fingers was significantly higher among dentists	Hand radiography	Dentists; Teachers	543	Dentists:54 ± 6; Teachers: 54 ± 4	F: 543	[15]
Spondylosis of the cervical spine in 52.1% of dentists and 19.2% of farmers	Radiologic examination	Dentists; Farmers	311	Dentists: 44.5 ± 20.5; Farmers: 44.5 ± 20.5	n.a.	[16]
Arthrotic distal interphalangeal (DIP) joints of all arthrotic joints of the hands was in both male and female dentists greater compared to controls	Hand radiography	Dentists	1076	51 ± 25.4	M: 487; F: 589	[17]
Degenerative cervical spine disease in 17%, rotator cuff pathology in 18%, degenerative lumbar spine disease in 19%, and carpal tunnel syndrome in 9%	n.a.	Physicians	5828	46	M: 4575; F: 1253	[6]
Higher femur BMD in the nurse group respect to the clerk’s occupational category	Dual-energy X-ray absorptiometry and osteocalcin assay	Nurses; Clerks	99	Nurses: 48.6 ± 7.6; Clerks: 47.2 ± 6.6	F: 99	[18]
		Farming sector				
Stanchion milking resulted in the greatest exposure to awkward knee posture (≥110° knee flexion)	Electrogonimetry	Dairy farm workers	24	42.8 ± 2.0	M: 24	[19]
Farmers working on farms with few animal contacts had a significantly lower risk of onset hip osteoarthritis	Physical and radiologic examination	Farmers	854	Farmers: 61.8 ± 6.3; Control group: 61.6 ± 6.2	n.a.	[20]
Musculoskeletal injuries of the knee are prevalent, especially among those with joint dysfunction (79.6%)	Clarke’s test, prone knee bending, straight leg raises, slump test, palpation test, and McMurray test	Rice farmers	201	45.3 ± 10.7	M: 86; F: 115	[21]
Women working in agriculture showed lower femur BMD compared to the control group	Dual-energy X-ray absorptiometry, 25(OH)D, and VDBP assay	Agriculture workers	390	Workers: 54.7 ± 9.2; Control group: 54.2 ± 7.7	F: 390	[22]
The prevalences of upper extremity diseases were 60.4% for rotator cuff tear, 20.9% for golfer’s elbow, 40.9% for tennis elbow, and 58.0% for hand osteoarthritis	Physical assessments, laboratory tests, radiographic examination, and magnetic resonance imaging	Fruit farmers	460	59.67 ± 8.04	M: 223; F: 237	[23]
		Industrial sector				
Radiological signs of osteoarthritis in the wrist and elbow joints were more frequent among the vibration-exposed workers	Radiologic examination	Foundry workers	113	39.6	n.a.	[24]
Decreased range of motion and osteoarthritis in relation to the duration of vibrating tool use	Radiologic examination	Stone quarry workers	74	51.1 ± 8.8	M: 74	[25]
No correlation between degenerative changes in the cervical spine and the duration of physical labor or exposure to hand-arm vibrations	Radiologic examination	Coal miners	685	46.3 ± 6.5	M: 685	[26]
The high-exposure group showed a higher prevalence of musculoskeletal complications, but no significant difference in the stratification of upper limbs and neck complications	Larson Davis HVM100 Human Vibration Meter	Tyre shop workers	200	31.9 ± 11.3	M: 200	[27]
Prevalence of lateral epicondylitis, De Quervain’s disease, and ulnar neuropathy of the elbow in coal miners	Physical examination and bilateral electrodiagnostic testing	Coal miners and clerical workers	123	Coal miners: 41.5 ± 4.9; Clerical workers: 41.9 ± 5.3	M: 123	[28]
Vibration represents a risk factor for osteoarthrosis of the acromioclavicular joint, but after simultaneous adjustment for manual work, this effect almost disappear	Radiologic examination	Bricklayers, rock blasters and foremen	207	Bricklayers: 50.2 ± 11.4; Rock blasters: 51.8 ± 11.6; Foremen: 45.8 ± 10.2	n.a.	[29]
Ligamentous outgrowths on the ventral beveling showed a statistically significant younger age in the robust group, indicating a possible faster rate or early timing of changes in individuals with more physically demanding activities	Visual assessments using a lamp light	Manual, non-manual, robust and gracile workers	161	57 ± 55.1	M: 161	[30]
Increased risk for posterior disc bulges among the carpenters and for anterior disc bulges among the machine drivers. Car driving was associated with anterior disc bulges	Magnetic resonance imaging	Machine drivers, construction carpenters and municipal office workers	164	42.5 ± 3.5	M: 164	[31]
Radiological evidence of disc degeneration in the lumbar spine was more frequent and severe in the foundry workers compared to controls	Radiologic examination	Foundry workers	325	54.5 ± 27.6	M: 325	[32]
Atrophied shoulder muscles were more common among welders than fishermen, whereas crepitations in the shoulder tended to be more common among fishermen	Orthopedic physical examination	Welders and office clerks	91	Welders: 41.1 ± 8.3; Office clerks: 41.2 ± 5.8	M: 91	[33]
Work involving kneeling and/or squatting is causally associated with an increased risk of osteoarthritis of the knee	n.a.	Coal miners	n.a.	n.a.	n.a.	[34]
Occupational kneeling increases the risk of degenerative tears in the medial but not the lateral menisci in both knees	Magnetic resonance imaging	Floor layers and graphic designers	141	56 ± 19.8	M: 141	[35]
Loads on the knees and the occurrence of minor injuries and accidents were higher in reinforcement work than in painting, but the occurrence of symptoms, clinical signs, and radiological findings was equally common in both groups	Physical and radiological examination	Reinforcement workers and painters	583	42 ± 31.1	M: 583	[36]
The femoral neck bone area was higher in beedi makers than in sweepers. The BMD of the lumbar spine was significantly lower among sweepers compared to beedi manufacturers. Sweepers and construction workers had a higher prevalence of osteoporosis in the lumbar spine	Dual-energy X-ray absorptiometry	Beedi (cigarette) makers, sweepers and construction workers	165	45 ± 21.2	F: 165	[37]
Vitamin D levels were associated with a progressive reduction in bone mineral density in the femoral neck and lumbar spine with 16% of subjects	Dual-energy X-ray absorptiometry	Garment workers	200	27 ± 12.7	F: 200	[38]
		Computer sector				
Cases showed reduced active wrist flexion compared with the control group, which did not appear to be related to pain	6″ universal goniometer	Computer workers	18	Computer workers: 43 ± 5.1; Control group: 46 ± 17	M: 7; F: 11	[39]
Symptomatic subjects tended to perform computer work with greater muscle load than non-symptomatic subjects	Electromyography	Computer workers	39	Computer workers: 28 ± 9; Control group:24 ± 2	n.a.	[40]
Air traffic controllers consistently had a higher prevalence of elbow–hand complaints in follow-up than those who did not use computers	Standardized physical examination	Air-traffic controllers	148	41 ± 24	M: 77; F: 71	[41]
Forearm muscles in symptomatic individuals were inhibited in their maximal activation as well as during functional tasks	Electromyography	Computer workers	17	Computer workers: 36.23 ± 5.40; Control group: 26.50 ± 3.02	n.a.	[42]

OA: osteoarthritis; BMD: Bone Mineral Density; VDBP: vitamin D-binding protein; n.a.: not applicable.

**Table 2:** Prevalent pathologies and likely causes in different employment sectors and related occupational categories.

Employment Sector	Occupational Category	Prevalent Pathology	Likely Cause
Healthcare sector	Doctors	Osteopenia	Sedentary life
			No time to devote to physical activity
			Poor eating habits
			Poor exposure to the sun
	Dentists	Hand osteoarthritis	Repetitive hand and arm movements
			Vibrations
			Poor posture of the neck and upper limbs
			Prolonged duration of movements
			Precision gripping
			Monotonous work activity
			Joint overuse
Farming sector	Farm workers with animals	Knee and hip osteoarthritis	Uncomfortable postures
			Lifting heavy loads
			Standing for a long period of time
	Growers	Osteoporosis	Sedentariness
	Fruit tree growers	Hand osteoarthritis	Maintaining a static position
			Lifting hands and arms for a prolonged time
			Working for more than 10 h a day
			Female sex
Industrial sector	Coal miners	Knee osteoarthritis	Uncomfortable postures
		Lateral epicondylitis, De Quervain disease, and ulnar neuropathy	Uncomfortable postures
			Vibrations
			Repetitive movements
			Lifting heavy loads
			Cold
			Mechanical power presses
	Costruction workers	Osteoporosis	Poor eating habits
		Atrophic shoulder muscles	Uncomfortable postures
		Lumbar disc degeneration	Lifting heavy loads
	Vibration-exposed workers	Osteoarthritis of the elbow, wrist, and acromioclavicular joint	Vibration
			Lifting heavy loads
		Musculoskeletal disorders of the upper limb and neck	Vibration
		Rheumatic diseases	Vibration
			High temperature
			Maintaining a static position
	Occupational Kneeling	Degeneration of the knee joints and alteration and tearing of the medial meniscuses	Uncomfortable postures
			Prolonged duration of movements
	Factory workers	Osteoporosis	Poor eating habit
			Poor exposure to the sun
Computer sector	Computer workers	Musculoskeletal disorders of the upper limb, neck, and shoulders	Distance of the device from the medial axis of the body
			Repetitive movements
			Muscle tension
			Reduced extension/flexion of muscles due to posture
			Increased activity of neck muscles

## Data Availability

Data will be made available on request.

## Supplementary Materials

The following supporting information can be downloaded at https://www.mdpi.com/article/10.3390/jcm13133964/s1, Table S1: PRISMA 2020 checklist.
